# Technical feasibility of online adaptive stereotactic treatments in the abdomen on a robotic radiosurgery system

**DOI:** 10.1016/j.phro.2022.07.005

**Published:** 2022-07-28

**Authors:** Maaike T.W. Milder, Alba Magallon-Baro, Wilhelm den Toom, Erik de Klerck, Lorne Luthart, Joost J. Nuyttens, Mischa S. Hoogeman

**Affiliations:** Erasmus MC, University Medical Center Rotterdam, Department of Radiotherapy, The Netherlands

**Keywords:** Adaptive, ART, SBRT, LAPC, Oligometastases, CyberKnife

## Abstract

**Background and purpose:**

Stereotactic body radiotherapy (SBRT) has been proven to be beneficial for several disease sites in the (lower) abdomen. However, the quality of the treatment plan, based on a single planning computed tomography (CT), can be compromised due to large inter-fraction motion of the target and organs at risk (OARs) in this anatomical region. The aim of this study was to investigate the feasibility of online adaptive SBRT treatments on a robotic radiosurgery system and to record estimated total treatment times.

**Materials and methods:**

For two disease sites, locally advanced pancreatic cancer (LAPC) and oligometastatic lymph nodes, four patients with repeat CTs were included in the feasibility study. Quick treatment plan templates were generated based on the planning CT and validated by running them on the plan and fraction CTs. For two cases a dummy run was performed and the individual steps were timed. Dose delivery was the largest contributor to the total treatment time, followed by contour adaptation.

**Results:**

Running the quick plan templates resulted in plans similar to unrestricted plans, obeying the OAR constraints. The dummy runs showed that online adaptive treatments were completed in 64 to 83 min respectively for oligometastases and LAPC, comparable to other clinically available solutions.

**Conclusions:**

This study showed the feasibility of online re-planning for two challenging disease sites within a clinically acceptable time frame on a robotic radiosurgery system, making use of commercially available elements that are not integrated by the vendor.

## Highlights

• Online adaptive radiotherapy is feasible on a robotic radiosurgery system.
• Plan templates for fast plan re-optimization were developed and successfully tested.
• Dummy runs were performed for two abdominal treatment sites.
• Total treatment times were 64 and 83 min respectively.

## 1 Introduction

In the past decade stereotactic body radiotherapy (SBRT) was shown to be beneficial for the treatment of primary tumours and oligometastases [1], [2], [3], [4]. SBRT has also been employed for targets in the abdomen. It has earned a place in the treatment of prostate, liver and pancreatic tumours and is increasingly used to treat abdominal oligometastases. In (upper) abdominal treatment sites the target and the radio-sensitive OARs show both respiratory and large non-respiratory motion, leading to a potential loss in treatment plan quality. A robotic radiosurgery system is well-equipped to manage both types of motion of target sites using real-time respiratory motion tracking. However, the motion of OARs can occur both inter- and intra-fractionally, and is less straightforward to handle, with the actual delivered dose to the OARs likely to be over- or underestimated compared to the planning CT. An additional challenge in abdominal sites is the vicinity of highly radiosensitive OARs to the target. As a result, the dose to the target can be compromised to fulfil the OAR dose constraints.

There are several, increasingly complex, techniques to improve the treatment of moving targets. However, the availability of techniques to manage moving OARs, especially in close proximity to the target, is still limited. Online adaptive radiotherapy (ART) with daily plan adaptation is a technique that does offer a solution to the inter-fraction motion of targets and OARs [5], [6], [7], [8], [9], [10], [11], [12]. Online ART is based on pre-fraction imaging after which the target and OARs are delineated and a new plan is generated. Whereas in the past mainly MRI and CT images were used for ART, this has started to change as online adaptive treatments based on enhanced CBCT images became commercially available.

Currently, the commercially available MRI- and CBCT-guided online ART solutions have limited options for intra-fraction motion management including compensation for respiratory motion. One of the available MRlinac systems employs gating [6], [13], [14], while a second MRlinac system and the current CBCT-guided systems require the use of a larger margin to compensate for respiratory motion of the target or the use of abdominal compression to try and minimize this motion [15], [16], [17], [18].

A combination of a CT scanner and a robotic radiosurgery system can offer a combination of intra-fraction motion management and online ART [19]. This offers state-of-the-art treatments for both targets and OARs showing inter- and intra-fraction motion. In previous work we have described a fast and effective way of making online adaptive plans [8]. In this paper we aimed to demonstrate, the feasibility of clinically implementing an ART SBRT workflow for two challenging treatment sites and record the estimated total treatment time. This workflow is currently not supported by an integrated, clinical product and was developed by our team using commercially available elements. A method that could be transferred to other treatment sites and clinics.

## 2 Material and Methods

### 2.1 Patient characteristics

Two groups of patients with challenging disease sites; inoperable locally advanced pancreas carcinoma (LAPC) and lymph node oligometastases, were included in this retrospective feasibility study for online adaptive SBRT. For each of these groups, four patients, previously treated in the Erasmus MC, with multiple fraction CT scans were included. A total of 12 and 18 repeat scans was available respectively for LAPC and oligometastatic patients for quick plan generation and evaluation. Due to clinical limitations one STEAL patient received only three repeat CT scans. Planning and fraction CT scans were acquired on a Sensation Open and a SOMATOM Definition AS CT scanner respectively (Siemens Healthcare, Forchheim, Germany). The CT Voxel size was 0.98 × 0.98 × 1.5 mm^3^. Patients with LAPC were treated in the LAPC-1 Phase-II study (ID: NL49643.078.14) and received 40 Gy in five fractions prescribed to the 80 % isodose line. Patients with lymph node oligometastases in the pelvis and abdomen were treated in the STEAL study (ID: NL58442.078.17) and received 45 Gy in five fractions prescribed either to the 80 or 90 % isodose line according to the study protocol. LAPC patients received a contrast-enhanced CT scan before the first three fractions and the patients with oligometastatic lymph nodes received a CT scan before each fraction (i.e. five in total). For both groups pre-fraction CT scans were obtained in treatment position using the CT on-rails while the robotic couch was moved between its in-room imaging and treatment position [19]. The LAPC and STEAL patients were treated on the Cyberknife M6 system (Accuray Inc., Sunnyvale, CA, USA) using fiducial tracking with real-time respiratory motion tracking and intra-fraction spine tracking respectively [20]. Patients included in this study have previously all given informed consent. The LAPC patients included in this work were used in a previous study, and were selected to reflect the range of PTV sizes [8], [21].

### 2.2 Offline planning CT delineation and plan generation

All delineations of target and OARs were performed and/or supervised by experienced radiation oncologists. The planning CTs were delineated in Precision (version 3.1), the Accuray treatment planning system (TPS).

**Fig. 1:**
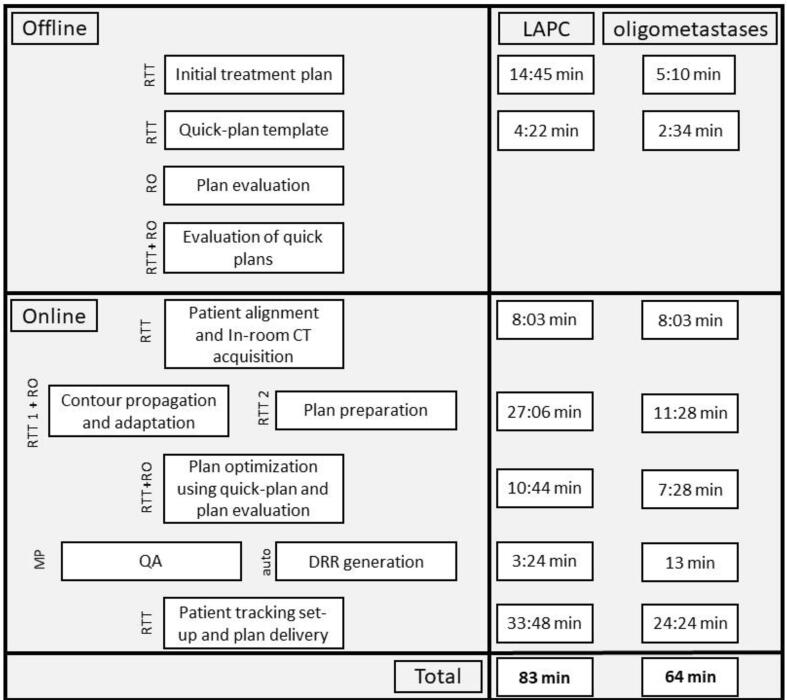
Offline procedure and the different steps of the online adaptive procedure. In the online procedure several steps occur in parallel. The respective timings of the individual steps is different for the two tumour sites. Patient set-up and 3D imaging was performed once. Contour propagation and adaptation was performed one time for both sites. Plan optimization and treatment set-up time and delivery are average times over the patients included in this study. The DRR generation is a population average for the respective tracking type.

For the two groups of patients treatment plans were generated by radiation technologists (RTT) in Precision, based on current clinical protocols. The VOLO optimizer for the MLC collimator was employed for both sites [22], [23], [24], [25]. The planning dose constraints can be found in the supplementary materials (Table S1). The resulting plans were approved by an experienced radiation oncologist. Based on the optimization parameters of this clinical plan a quick-plan template was derived for use in the online procedure [8] (Fig. 1). This quick-plan was dosimetrically equivalent to the clinical plan but was further adjusted to reduce the optimization time, including full fluence optimization and segmentation. The quick-templates were achieved by truncating the OARs structures 2 cm from the PTV for optimization purposes and by reducing the sampling of the dose limiting structures, shells, and the target to get a total number of optimization points <50,000 [8], [26]. The truncation of the OARs was for optimization purposes only and the planned dose was evaluated on the complete structures. The shells were used to limit the dose to the surrounding tissues.

### 2.3 Offline quick plan evaluation

To evaluate the efficacy of the patient-specific quick-templates, run after manual plan preparationon the available fraction CT scans, the resulting dose was directly evaluated without further human intervention. Relevant DVH parameters (Table S1 supplementary materials) were extracted and compared to the clinical dose constraints to establish if a clinically acceptable plan was generated. The dose was evaluated on the complete OARs. For this purpose the fraction CTs were delineated in MIM (version 6.9), supervised by an experienced radiation oncologist.

### 2.4 Dummy runs

For both tumour sites a dummy run was organized to evaluate the feasibility of an online adaptive treatment with the available resources. During these dummy runs, the complete clinical team, two RTTs, a radiation oncologist and a medical physicist, was present and ran through the procedure with a phantom, while timing the individual steps. The goal of these dummy runs was to give an indication of the time required for each individual step of the online procedure and identify bottlenecks and areas of improvement. Where possible, steps were performed in parallel to minimise the total time.

### 2.5 Online ART procedure

The online procedure is schematically represented in Fig. 1. Both online procedures entailed the following steps: (1) patient alignment and in-room CT acquisition, (2) contour propagation and adaptation, (3) plan preparation, optimization and evaluation, (4) patient specific quality assurance (QA) and generation of digitally reconstructed radiographs (DRR) and (5) patient tracking set-up and plan delivery. To simulate patient set-up and image acquisition, a phantom was positioned on the robotic couch in treatment position. Subsequently, the table was moved to the scan position and CT images were acquired [19]. To mimic a real patient procedure, a CT scan of a patient was used in the dummy runs instead of the acquired phantom scan. The scan belonged to a patient for whom a quick-plan template was prepared offline and was forwarded for delineation in MIM (version 6.9). For both treatment sites deformable image registration was used to transfer OAR contours from the planning CT to the fraction CTs, while the target was copied rigidly. This rigid transformation was based on a fiducial match for LAPC and a match on the vertebrae for lymph node metastases, to mimic the intra-fraction tracking method. A radiation oncologist edited contours within a 3 cm ring from the PTV while in parallel an RTT was preparing the treatment plan up to the point that a contour set was required. Subsequently, the adapted contours were transferred to the TPS and the prepared quick-plan template was run. The radiation oncologist evaluated the plan while the medical physicist started patient specific QA. This step ran in parallel with plan DRR generation. The final steps of the procedure were patient tracking set-up and plan delivery. Patient-specific quality assurance was executed in the form of an independent 3D Monte Carlo recalculation using SciMoCa (scientific RT) [27].

## 3 Results

Table 1 shows the optimization times and dosimetric parameters of the clinical plans and the quick-templates. It shows that the optimization time in a quick plan was reduced by a factor of 3.4 and 2.2 for LAPC and lymph nodes metastases respectively, without compromising the plan quality. The latter was evaluated by comparing the relevant DVH parameters of the unrestricted and the quick plan of all available planning CTs. As reported previously these parameters were similar and the small differences mostly insignificant . The largest differences were observed in the shapes of the DVH of the OARs, while the mean dose remained similar. Dose distributions and the DVH of two cases for each target site are shown in the supplementary materials (Figs. S1 and S2).

**Table 1:** Offline and online average optimization time and range in min, the number of plans within dose constraints and relevant DVH parameters for the plan comparison. The values of the DVH parameters for the clinical unrestricted and the quick plan are averaged over all planCTs.

	Clinical unrestricted plan		Quick plan	
Offline Optimization Time planCT (min:s), lymph nodes|LAPC	5:10 (1:48–15.06)	14:45 (5:14–30:20)	2:34 (1:14–5:37)	4:22 (3:28–5:09)
Online Optimization Time FxCT (min:s), lymph nodes|LAPC			2:42 (1:21–5:39)	3:28 (2:28–4:11)
FxCT plans within all dose constraints, lymph nodes|LAPC	4/4	4/4	18/18	12/12
PTV coverage (%)	93.0		92.8	
GTV coverage (%)	98.3		98.6	
PTV CI	1.09		1.09	
PTV mean (Gy)	45.6		45.5	
GTV mean (Gy)	47.1		47.1	
Mean dose OARs (Gy)	6.1		6.3	
OARs D0.5 cm^3^ (Gy)	30.2		29.7	

Using the quick templates for plan optimization in the fractions resulted in plans that fulfilled the dose constraints in all cases. In several fractions the OARs moved closer to the targets and in some cases even overlapped with the PTV. To fulfil the OAR constraints in these cases the PTV coverage was compromised, the average PTV coverage was 1.7 vs 2.4 % lower for LAPC and lymph nodes respectively. The coverage of the GTV remained more constant with a 1 % decrease for LAPC and a 0.2 % increase for oligometastatic lymph nodes. Tables S2 and S3 in the supplementary materials show the extracted DVH parameters for the individual patients.

Fig. 1 shows the major steps of the dummy runs with their respective timings. The total adaptive treatment time was 83 min for LAPC and 64 min for oligometastases. Patient set-up and 3D imaging contributed roughly 10 % (8 min). Subsequently, propagating the contours from the planning CT to the fraction CTs and manually editing them was responsible for a considerable amount of the total treatment time up to 18–32 % (i.e. 11 and 27 min for oligometastases and LAPC respectively). This difference was explained by the number of OARs involved: for LAPC three OARs (duodenum, stomach and bowel) require editing, while for oligometastases usually only one critical OAR needed reviewing. In addition, the target in LAPC was often larger and more complex to delineate. Plan generation and evaluation took 12–13 % of the total treatment time (7 and 11 min for oligometastases and LAPC respectively). DRR generation time was highly dependent on the tracking method used. For the LAPC patients using fiducial tracking this accounted for only 4 % (3.5 min) of the total, while for the spine tracking method used for oligo metastatic lymph nodes this increased to 20 % (13 min). With 4–5 min, QA occurred in parallel to the DRR generation and patient tracking set-up. The largest contributor to a simulated online adaptive treatment fraction was patient tracking set-up and treatment delivery (33 and 24 min for oligometastases and LAPC respectively, including 5 min tracking set-up around 40 % of the total). The treatment delivery time was obtained by averaging over all patients for the two different treatment sites.

## 4 Discussion

This is the first study that describes the development and tests the feasibility of online adaptive SBRT on a robotic radiosurgery system using in-room CT imaging and commercially available software elements not integrated by the vendor. The dummy runs demonstrate the delivery of online adaptive SBRT in this setting with an in-room treatment time of 64–83 min, about 2.5 times that of a standard fraction delivery and comparable to other current online adaptive SBRT techniques [6], [17], [28]. Robotic radiosurgery treatments are inherently longer due to tracking and non-coplanar dose delivery. The treatment delivery time increases with target size and treatment complexity, from 19 min for lymph nodes (average PTV size 32 cm^3^) to 29 min (average PTV size 139 cm^3^) for LAPC.

Different aspects in the online procedure can still be optimized. One time-consuming steps is the deformable image registration and contour propagation in the abdomen and pelvis [8], [29]. The contours of the DIR algorithms remain inaccurate and laborious editing is required in most cases. Especially in the case of LAPC patients, due to the close vicinity of three large OARs and a large and complex target, contour editing takes up a considerable amount of time. Even when editing was restricted to 3 cm around the target, 20 min was needed for the target and OAR contours. Since then we have tested a strategy that divides the delineation, done in parallel by RTT (OARs) and radiation oncologist (target). In this way we could reduce editing to 10 min, similar to other reported times [10], [26], [28], [30], [31], [32]. A selected group of RTTs in our institute has already been trained to delineate OARs on planning CTs. They can therefore perform this role during the online procedure. A recent publication shows that this role differentiation is feasible, potentially even for a selected group of target sites [33], [34], [35]. In addition, technical solutions such as system integration and the use of AI have shown to shorten the time required for an online procedure [26], [30].

In cases with a larger target and or multiple OARs included in the optimization, i.e. with a large number of sample points, the standard optimization time can take up to 30 min. With the simple measures included in the quick plan, this time was greatly reduced. A downside is that during treatment preparation, until we have gained more experience, two plans are required: a clinical plan and a plan with reduced optimization time to assure the plan quality. By reducing the number of optimization points, the clinical and quick plan will never be completely identical and experience will have to show if we can omit the clinical plan as is suggested by this and previous studies. The efficacy of the quick plan template was demonstrated in a larger patient cohort for LAPC [8].

A limitation to our study is the limited soft-tissue contrast CT images can offer compared to MRI. Oligometastatic lymph nodes and their surrounding OARs are in general visible on CT images. These targets are therefore excellent candidates for (CB)CT-based online adaptive treatments. However, in (CB)CT-based ART for LAPC the limited soft-tissue contrast can restrict the visualization of the target. In our institute fiducial markers are implanted in the pancreas to ensure accurate intra-fraction target tracking and therefore allow the use of small PTV margins. Intra-fraction motion management is at the moment only available for one of the MRlinac systems in the form of gating. Hence, treatments on other systems currently require an ITV and or an extra PTV margin to compensate for the lack of intra-fraction motion management.

A major limitation, present in our workflow and most commercial solutions, is the time patients spend on the treatment table. Especially for the combination of (extreme) hypofractionation and highly mobile OARs, showing stochastic motion, this remains a problem: plan adaptations are already obsolete before they are delivered. Publications on intra-fraction motion of OARs remain scarce and are mainly restricted to the effect of rectum and bladder filling and peristalsis on intra-fraction prostate motion [18], [36], [37], [38]. Currently, in several institutes a virtual couch shift is applied at the end of full plan adaptation, based on a newly acquired 3D image [39]. The robotic radiosurgery system inherently offers this solution for target alignment correction at the start and throughout treatment delivery. One step further, real-time plan adaptation during a treatment fraction is explored as a solution for intra-fraction motion [40], [41], [42]. The effect of the table translation from imaging to the treatment position and further intra-fraction anatomical changes cannot be accounted for in our current clinical configuration.

At the moment the clinical implementation of online ART on a robotic radiosurgery system is limited by the time required for generation of DRRs used during intra-fraction motion management. For fiducial tracking this process takes about 3 min during which patient specific QA can take place. However, in case of spine tracking, in our institute used for the majority of lymph node oligometastases as they show little or no respiratory motion, this time increases to 13 min [43]. In the scope of a total in-room time that we aim to keep below 60 min, this leads to an unacceptable increase.

With this work we have shown that it is feasible to deliver online adaptive SBRT using re-planning with the robotic radiosurgery system, however there currently is no integrated software solution. Based on published experiences with dedicated systems for adaptive treatments [26], [30], [44] we expect the workload to decrease, the efficiency to increase and the total time required for the procedure to decrease.

In conclusion, this is the first report on the development of online adaptive SBRT using plan re-optimization on a robotic radiosurgery system with an in-room CT on rails. A dummy run showed the feasibility of this technique for two challenging disease sites (LAPC and lymph node oligometastases) within a clinically acceptable time frame, using commercially available, non-integrated components. Therefore, this technique can be transferred to other clinics and treatment sites. We are currently working on introducing this technique in our clinic for the SBRT treatment of high-risk prostate cancer.

## Declaration of Competing Interest

The authors declare the following financial interests/personal relationships which may be considered as potential competing interests: The Erasmus MC Cancer Institute has research collaborations with Elekta AB, Stockholm, Sweden and Accuray Inc, Sunnyvale, USA, and Varian, Paolo Alto, USA.
