# Supplementary material for: Technical feasibility of online adaptive stereotactic treatments in the abdomen on a robotic radiosurgery system
Source: Phys Imaging Radiat Oncol. 2022 Jul 28;23:103–8. doi: 10.1016/j.phro.2022.07.005 (PMC9344339; doi:10.1016/j.phro.2022.07.005)
Supplement: Supplementary data 1 [file mmc1.docx]

Supplementary Materials:

Table S1: Dose constraints and prescription isodose line for LAPC and oligo-metastatic lymph nodes

| Structure | LAPC (40 Gy) | Lymph nodes (45 Gy) |
| --- | --- | --- |
| PTV | 95% > 40 Gy | 95% > 45 Gy |
| Bowel | V35 Gy < 0.5 cm^3^ | V35 Gy < 0.5 cm^3^ |
| Duodenum | V35 Gy < 0.5 cm^3^ | V35 Gy < 0.5 cm^3^ |
| Kidneys |  | V15 Gy < 0.33% |
| Liver |  | 17.5 Gy < 700 cm^3^ |
| Skin |  | V35 Gy < 0.5 cm^3^ |
| Spinal Cord |  | 27.5 Gy < 0.5 cm^3^ |
| Stomach | V35 Gy < 0.5 cm^3^ | V35 Gy < 0.5 cm^3^ |
| Prescription isodose line | 80 % | 80 or 90 % |

Table S2: Summary for oligometastatic lymph nodes of the dosimetric DVH parameters and optimization times for the unrestricted and quick optimizations on the planning CT (pCT and pCT quick respectively). The fraction CTs (1 through 5) show results obtained after running a quick plan template.

Table S3: Summary for LAPC of the dosimetric DVH parameters and optimization times for the unrestricted and quick optimizations on the planning CT (pCT and pCT quick respectively). The fraction CTs (1 through 3) show results obtained after running a quick plan template.

Figure S1: Examples of dose distributions for the unrestricted and quick plans for oligometastatic lymph nodes (row 1 and 2) and LAPC (row 3 and 4) on the planCT.

| Patient 1: oligometastatic lymph node, left: unrestricted plan, right: quick plan |
| --- |
| 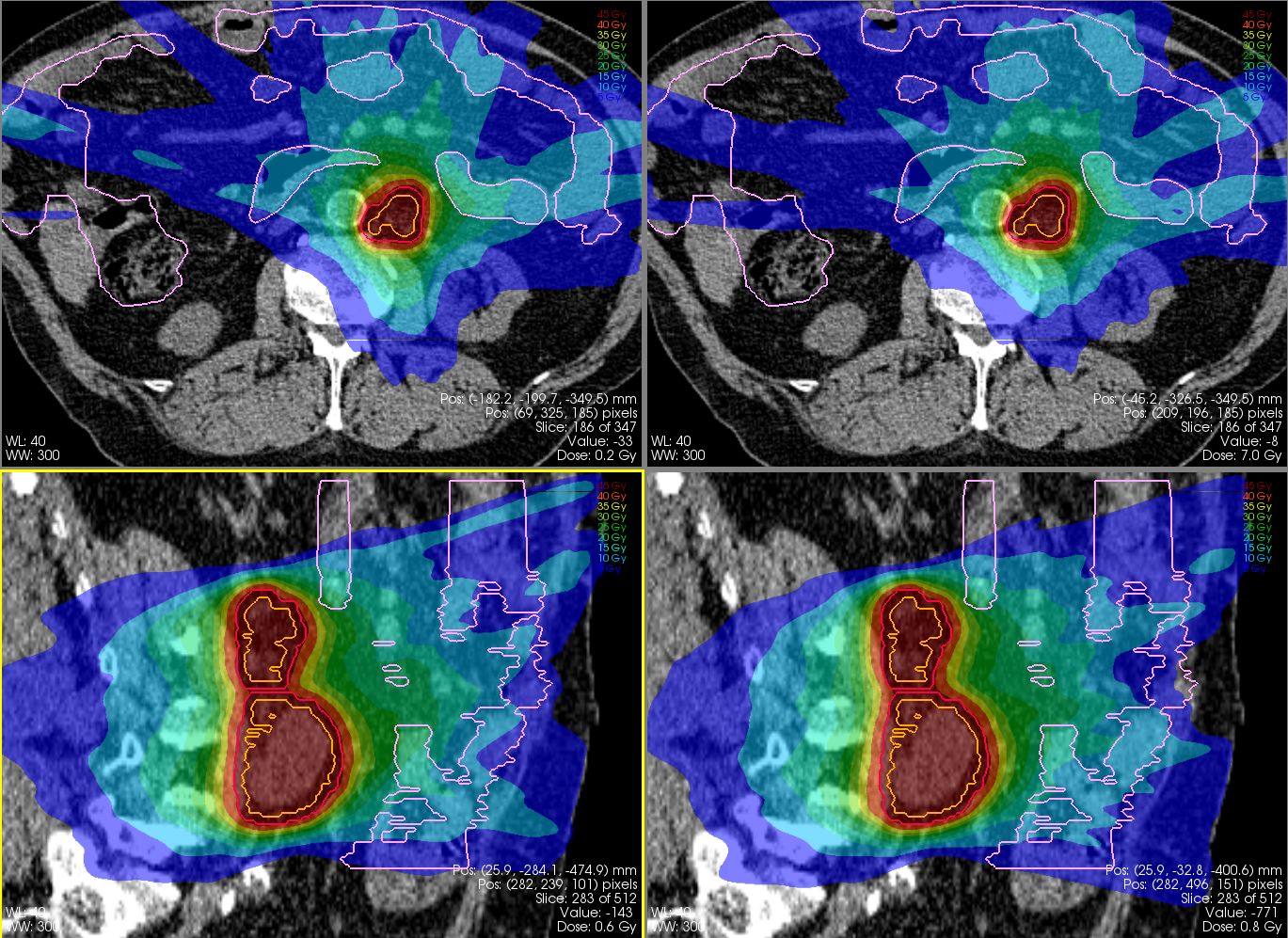 |
| Patient 4: oligometastatic lymph node, left: unrestricted plan, right: quick plan |
| 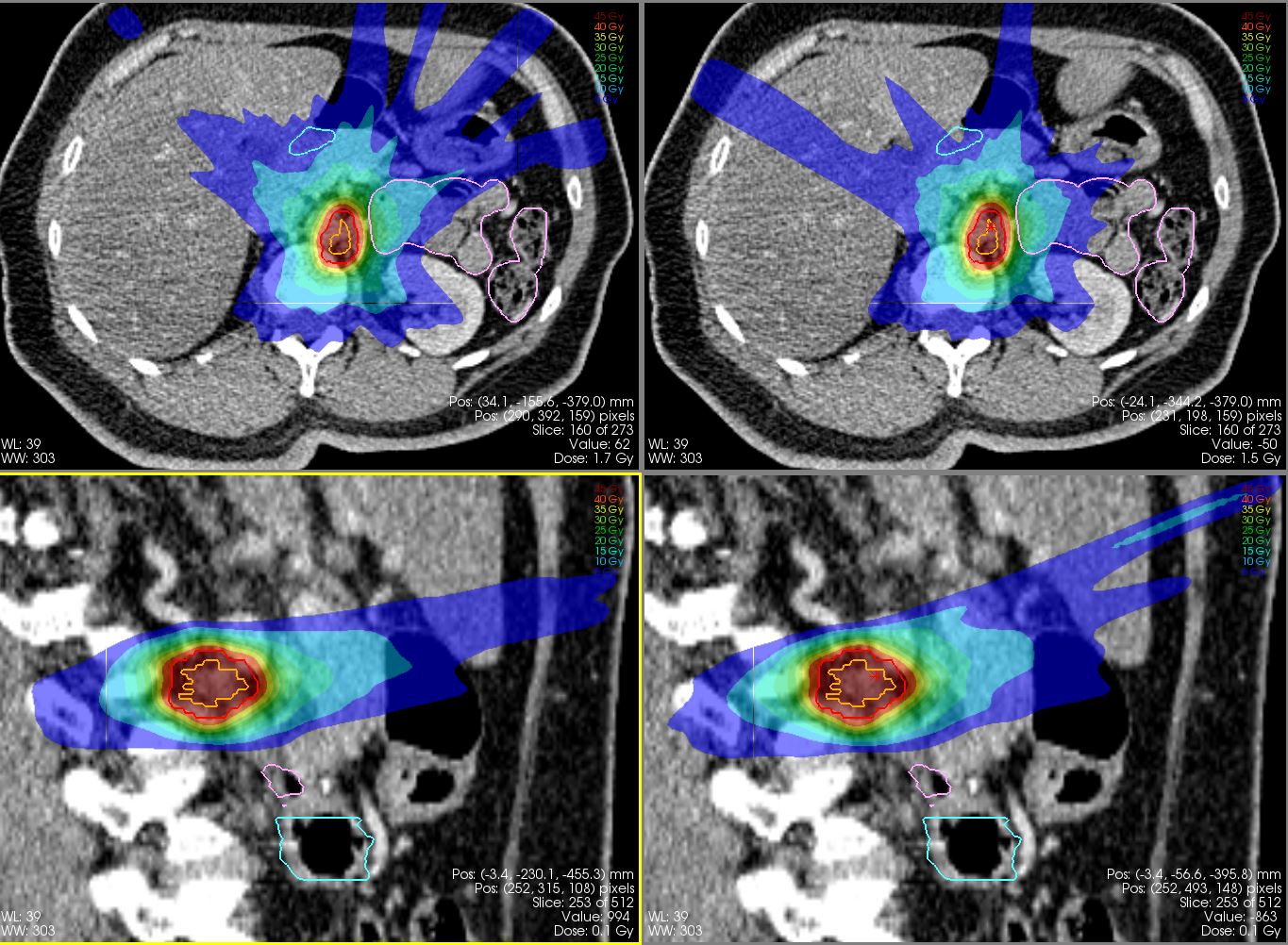 |
| Patient 5: LAPC, left: unrestricted plan, right: quick plan |
| 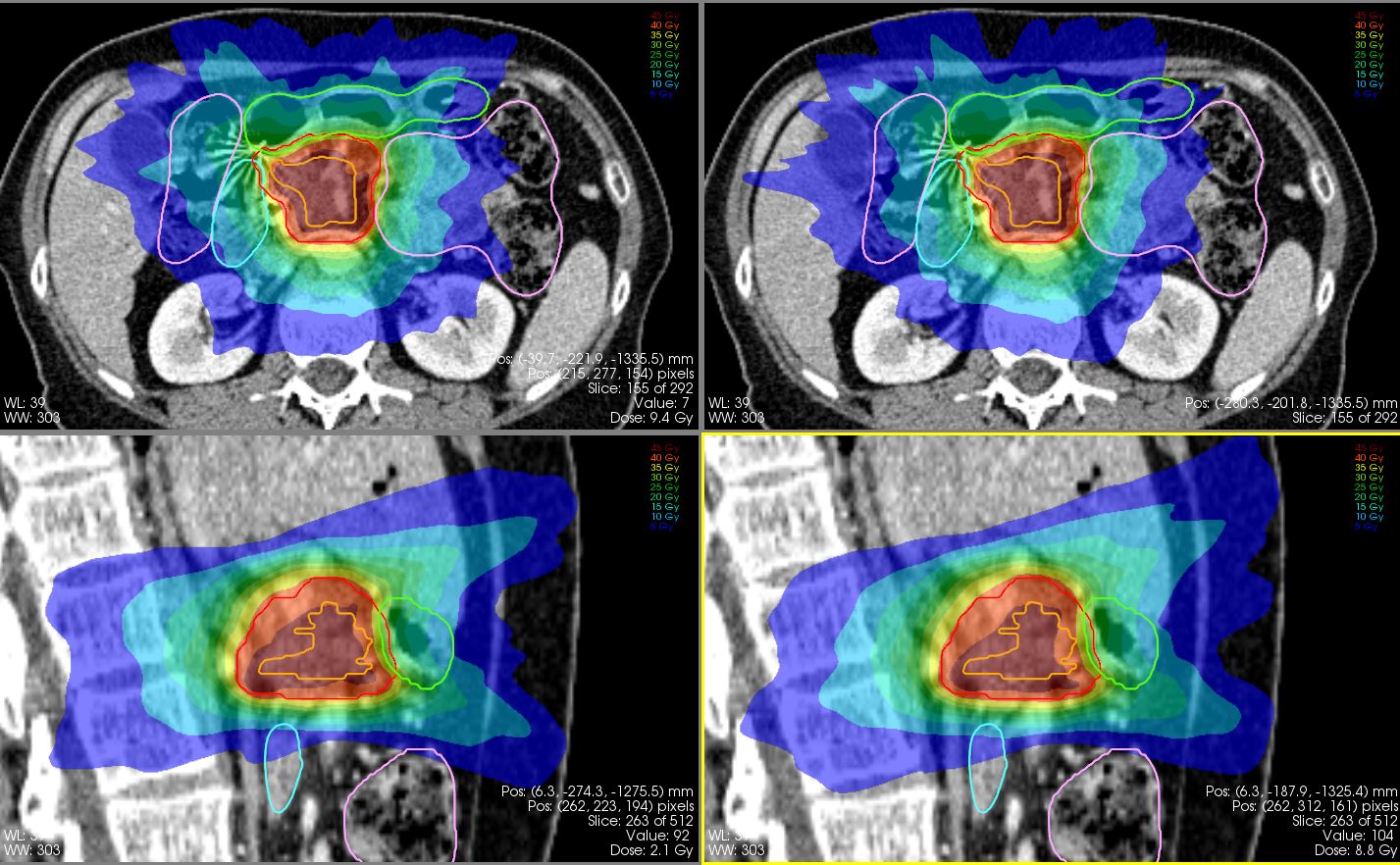 |
| Patient 8: LAPC, left: unrestricted plan, right: quick plan |
| 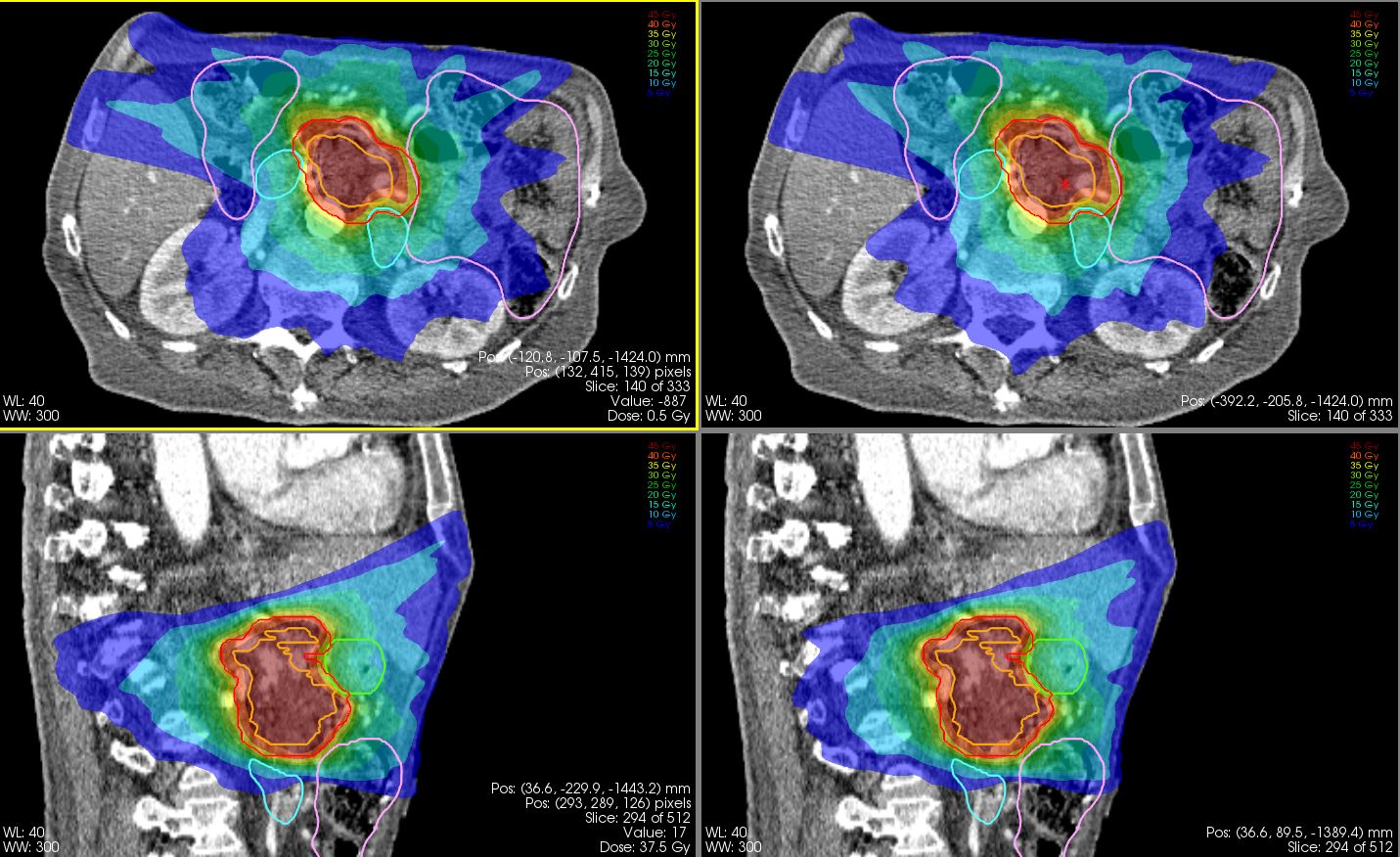 |

Figure S2: DVH views for the unrestricted and quick plans for oligometastatic lymph nodes (row 1 and 2) and LAPC (row 3 and 4) on the planCT.

| Patient 1: lymph nodes, solid: unrestricted plan, dash: quick plan |
| --- |
| 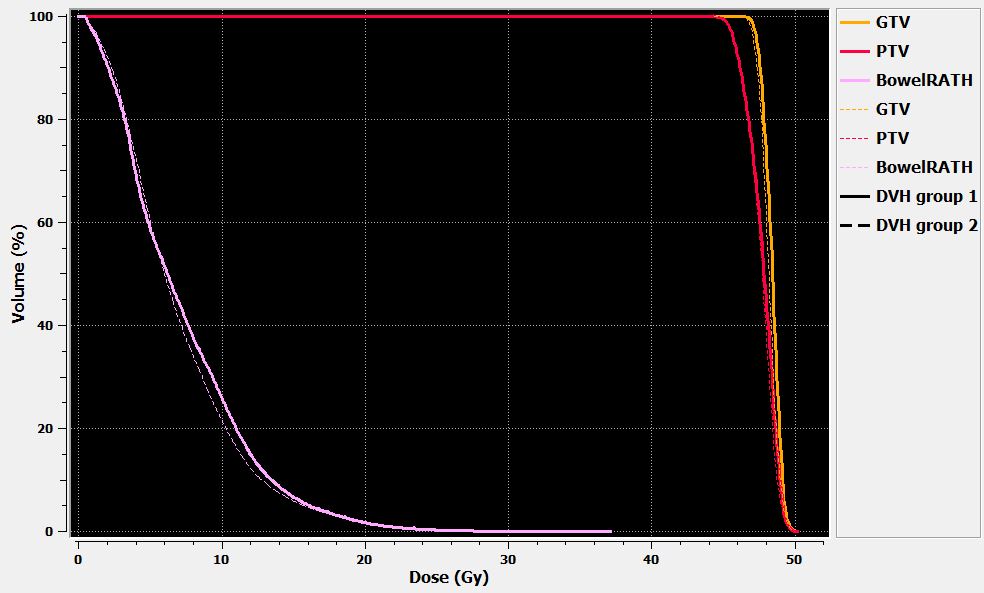 |
| Patient 4: lymph nodes, solid: unrestricted plan, dash: quick plan |
| 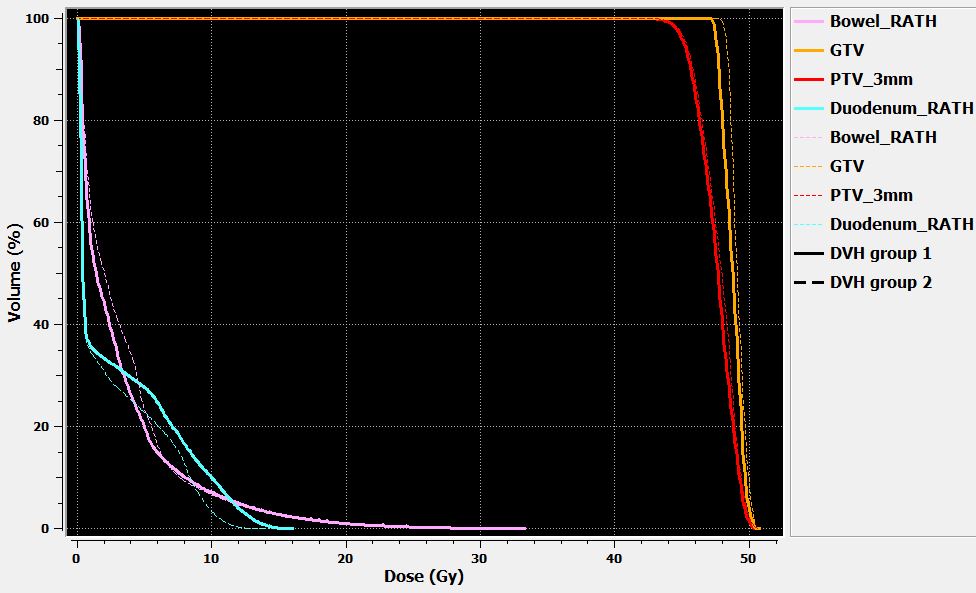 |
| Patient 5: LAPC, solid: unrestricted plan, dash: quick plan |
| 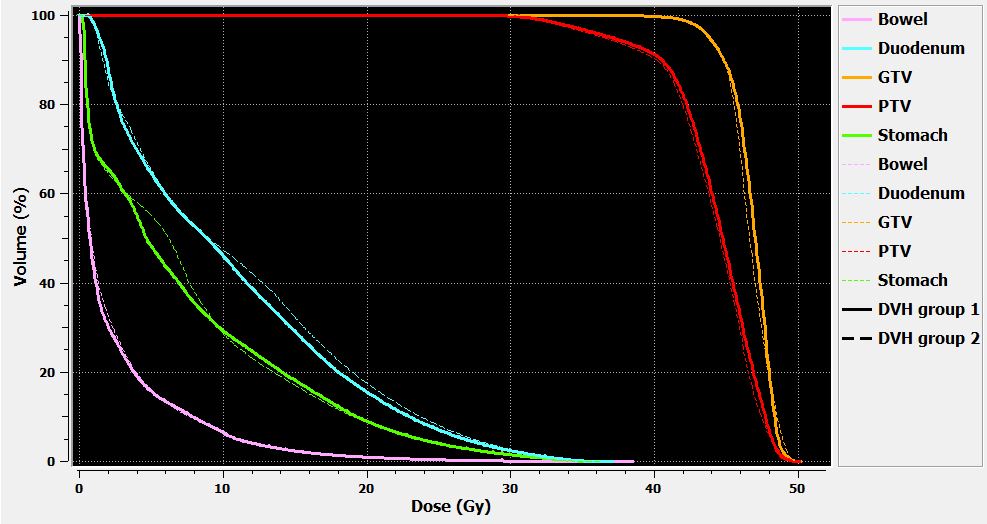 |
| Patient 8: LAPC, solid: unrestricted plan, dash: quick plan |
| 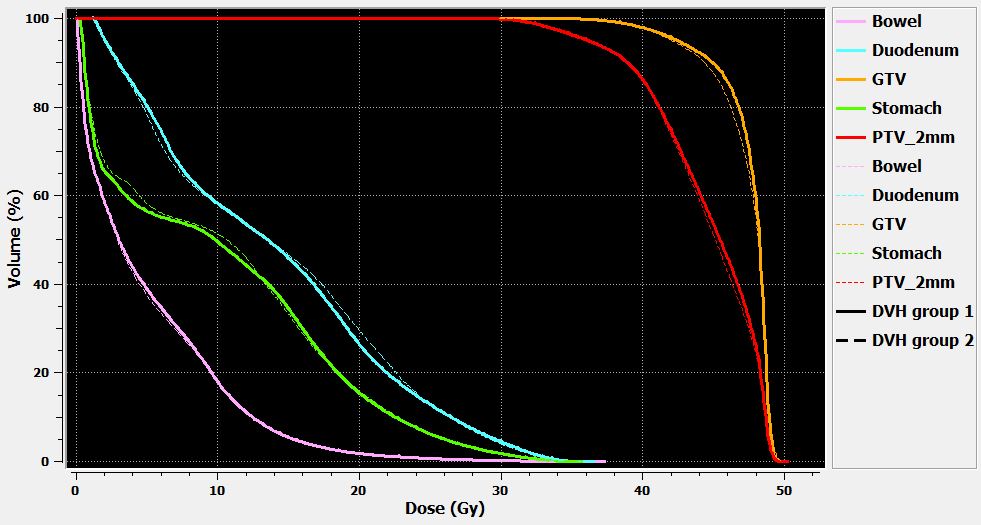 |
